# The Growth of the Nursing Profession: IX. Some War Thoughts and a New Spirit

**Published:** 1918-05-25

**Authors:** 


### THE MATRONS' AND SISTERS' DEPARTMENT.

### THE GROWTH OF THE NURSING PROFESSION.

### IX. Some War Thoughts and a New Spirit.

When a great movement such as that which has established the College of Nursing comes into being, and the unwonted stir which accompanies all new life throbs in the pulses of the community, then there is felt by those who instinctively send their thoughts winging a flight into future years something of the expectancy which prompted the exclamation in the early nineteenth century, "Bliss was it in that dawn to be alive, but to be young was very heaven." A strange sentiment to quote in these days of stress and strain, of unparalleled hardships, sufferings, and endurance. True, but how often has it not been proved that the darkest hour is that before the dawn! The hour may be prolonged, and those who at the beginning of the Great War were already leaving their youth behind may feel their consciousness so seared with unforgettable impressions of horror that never again will there come to them that true *joie de vivre* which in normal times is not confined to care-free youth, but springs irrepressibly in all hearts from which God's image has not been completely effaced. For the majority of us who have lived through this war there may remain only the calm and sober joy that comes from selfless work patiently and faithfully accomplished.

But it is when we pause and look round on this work that is being thus accomplished that we are conscious of the stirrings of a new spirit, and realise that when time has healed the wounds of this generation, there will be for the coming generations a future bright with hope, the dawn of a long-delayed fulfilment. It is stirring everywhere throughout our Empire, though perhaps some of its forms may seem strange and ill-omened to our weaker sight. In the nursing world, however, he would indeed be dull of understanding who could not discern the promise. We said in the first of these articles that nursing and all things connected therewith were in the melting-pot. The old order changeth, yielding place to new, and God fulfils Himself in many ways. Old traditions, hard-and-fast lines, narrow conceptions, ideas good in themselves, but unsuited to modern conditions—it was needful that these should go; but in order to get rid of them it has also been necessary to cast in much that is good; all, in fact, that has gone to create the nursing profession and to produce the women who to-day are standing at the post of duty with the same unflinching courage as the bravest of Britain's sons. What is to be the result? Is it not "up to" the nurses of this generation to see that that melting-pot shall be no mere seething cauldron casting up an incessant froth, but a veritable crucible out of which the gold shall come purified and refined from all alloy?

In these articles we have touched but lightly on many great questions that need to be solved before the nursing profession can take its right place in the world, and we have indicated various lines along which the College of Nursing might look for the solution. But we have kept always in mind this cardinal point—that the College of Nursing is no self-contained organism standing above and apart from the profession and dictating to the latter. It belongs to the nurses, it is in the hands of the nurses, and its future can be defined by no one but the nurses. Nor has it sprung full-grown from Jove's head. It has proved itself endowed abundantly with the power of growth and of assimilation, and herein lies its true greatness and power of appeal. Frequently the argument is brought forward that only by putting definite and immediate material attractions before nurses can they be induced to join any organisation, and that the ideal of the Trades Union—if ideal it can be called—is, if not the only one, yet certainly the most potent in its appeal to the majority of nurses. The 8,500 voting-papers sent out for the election of the College Council should be proof enough that this argument cannot be wholly true.

For the College of Nursing is sending forth a clarion call to the nurses of this generation, knowing that as the trumpet which summoned them to the succour of the wounded did not sound in vain, so neither will this other call awaken empty echoes, though it appeal neither to heroism nor to sentiment. What has posterity done for us? Because we must answer "Nothing," the call to nurses to join the College and mould the future of their profession has all the more force as appealing to something in them which is more enduring than themselves. It is a trite enough phrase—"the future of their profession"; we need to realise that it means the nurses of the future, the women that they will be, the work that they will do, and the part that they will play in eradicating disease and suffering from the world. We are confident that the nurses of this generation will quickly realise the innate worthiness of the work they are called to and will unite in the steadfast purpose of building up by patient endeavour and faithful co-operation, and with undimmed vision of fulfilment, the edifice of pure gold of which the material building bearing the name of the College of Nursing shall be but the outward and visible sign.

☛ Previous articles appeared Jan. 19, 26; Feb. 9, 23; March 16; and April 6, 20 and 27, p. 81.

